# River Dolphins Can Act as Population Trend Indicators in Degraded Freshwater Systems

**DOI:** 10.1371/journal.pone.0037902

**Published:** 2012-05-29

**Authors:** Samuel T. Turvey, Claire L. Risley, Leigh A. Barrett, Hao Yujiang, Wang Ding

**Affiliations:** 1 Institute of Zoology, Zoological Society of London, Regent’s Park, London, United Kingdom; 2 University of Liverpool, Leahurst Campus, Neston, Cheshire, United Kingdom; 3 Freshwater Dolphin CIC, Madrid, Spain; 4 Key Laboratory of Aquatic Biodiversity and Conservation, Institute of Hydrobiology, Chinese Academy of Sciences, Wuhan, China; Leibniz Center for Tropical Marine Ecology, Germany

## Abstract

Conservation attention on charismatic large vertebrates such as dolphins is often supported by the suggestion that these species represent surrogates for wider biodiversity, or act as indicators of ecosystem health. However, their capacity to act as indicators of patterns or trends in regional biodiversity has rarely been tested. An extensive new dataset of >300 last-sighting records for the Yangtze River dolphin or baiji and two formerly economically important fishes, the Yangtze paddlefish and Reeves’ shad, all of which are probably now extinct in the Yangtze, was collected during an interview survey of fishing communities across the middle-lower Yangtze drainage. Untransformed last-sighting date frequency distributions for these species show similar decline curves over time, and the linear gradients of transformed last-sighting date series are not significantly different from each other, demonstrating that these species experienced correlated population declines in both timing and rate of decline. Whereas species may be expected to respond differently at the population level even in highly degraded ecosystems, highly vulnerable (e.g. migratory) species can therefore display very similar responses to extrinsic threats, even if they represent otherwise very different taxonomic, biological and ecological groupings. Monitoring the status of river dolphins or other megafauna therefore has the potential to provide wider information on the status of other threatened components of sympatric freshwater biotas, and so represents a potentially important monitoring tool for conservation management. We also show that interview surveys can provide robust quantitative data on relative population dynamics of different species.

## Introduction

Charismatic large vertebrates represent a very small component of global biodiversity, but have always received a disproportionate amount of conservation attention and funding due to high public and scientific interest in such species [1]. Although the conservation focus on large vertebrates may be partly justified because these species are particularly vulnerable to extinction in comparison to many other taxa [2], the limited resources available for conservation mean that this bias is often defended by suggestions that it will provide benefits to wider levels of biodiversity. Because they usually require relatively extensive geographical areas to support viable populations, large vertebrates (typically large mammals and birds) are regularly used as focal ‘umbrella’ species in protected area development and management, on the assumption that appropriate protection will also be conferred to sympatric co-occurring species assemblages [3]–[4]. It is also suggested that there may be a direct ecological link between the presence of large vertebrates (both carnivores and herbivores) and high levels of biodiversity, because they are either ‘keystone’ species that directly promote high biodiversity (e.g. through resource facilitation or trophic cascades), or ‘indicator’ species that are ecologically constrained to be spatially and temporally associated with high biodiversity [5]–[7]. Clarifying the potential usefulness of large vertebrates as monitoring tools for sympatric biodiversity is therefore an important priority in conservation research, especially for species-rich but highly threatened habitats such as freshwater systems.

However, the ability of large vertebrates to act as surrogates for wider biodiversity has been increasingly questioned. There is little quantitative evidence that these taxa are actually efficient umbrella, keystone or indicator species [4], [6], [8]–[12], suggesting that they may have limited usefulness or relevance as monitoring tools in a wider conservation context [7], [13]. Previous studies have also not investigated temporal changes in the population status of large vertebrates and other sympatric species as environments become progressively modified through human agency, to assess whether population trends are correlated between species and whether large vertebrates can act as “population trend indicators” rather than “classic” indicator species (i.e. species associated spatially rather than temporally with other taxa of interest/concern, although other definitions also exist [14]). In freshwater systems, recent studies have highlighted the potential for macroinvertebrate taxa (e.g. molluscs, aquatic insects) to act as surrogates in broader-scale biodiversity assessments [15]. In contrast, mammals, birds and amphibians have been shown to be poor surrogates for patterns of both richness and threat in wider freshwater diversity at a continental level [16]. However, the capacity for specific large freshwater vertebrates (typically apex predators with large energetic requirements [17]) to act as potential indicators of patterns and trends of regional biodiversity in freshwater systems has been the subject of very little rigorous investigation. The role of river dolphins as potential indicator species or population trend indicators is particularly important, because most Asian river systems in which these species occur contain extremely high levels of endemic biodiversity and provide essential ecosystem services in terms of food security and water availability for a considerable proportion of the world’s human population, but are increasingly threatened by extensive habitat degradation [18]. However, the only available quantitative study conducted so far on the relationship between Asian river dolphins and freshwater fish diversity suggests that Ganges River dolphins (*Platanista gangetica*) may be resilient to changes in fish community structure, and their status may not provide an accurate indication of the depletion of specific fish resources [19]. Further studies are therefore required to clarify the potential for charismatic large vertebrates such as river dolphins to monitor status and trends of freshwater biodiversity in threatened aquatic systems.

The Yangtze River drainage, the longest river system in Asia, supports approximately 10% of the world’s human population [20], and has been seriously affected by overexploitation and industrialization. The river’s fisheries have experienced severe declines [21]–[23], and two former economically important fish species may now be regionally or globally extinct. The Yangtze paddlefish (*Psephurus gladius*) is a diadromous migratory species that was restricted to the Yangtze drainage and neighbouring East China Sea by the late twentieth century, although it was also historically recorded from the Yellow River and Yellow Sea [24]. This species was widely targeted in the past for food [25]–[26], but it is now considered to be Critically Endangered (Possibly Extinct) by the IUCN [27]. Another fish species, Reeves’ shad (*Tenualosa reevesii*), constituted one of the most important commercial fisheries in the middle-lower Yangtze until the mid-1970s, but the shad fishery closed in 1987, and the species is also now probably extinct in the Yangtze [28]–[30]. The Yangtze ecosystem also contains two endemic cetaceans, the Yangtze River dolphin or baiji (*Lipotes vexillifer*) and the Yangtze finless porpoise (*Neophocaena asiaeorientalis asiaeorientalis*). Both of these apex predators have also experienced serious declines during recent decades due to incidental by-catch in fishing gear, vessel strikes, and wider-scale habitat degradation [31]–[32], and the baiji is probably now extinct, representing the first global large mammal species extinction in over 50 years [33]. The Yangtze paddlefish, probably the world’s largest freshwater fish, is a truly megafaunal species that may have reached 7 metres in length [34] and did not breed before 8–12 years of age [24], whereas Reeves’ shad only reaches 70 centimetres and spawns by 2–5 years of age [28]. Baiji have intermediate values for these parameters, with a maximum body length of c. 2.5 metres and reaching sexual maturity at 4–6 years of age [35].

Although some historical catch records or abundance estimates are available for each of these species [24], [28], [33], these data were collected in different ways and have relatively little temporal overlap. In particular, the pattern of the baiji’s historical decline has been difficult to interpret due to substantial variation in survey effort and methods, and a lack of quantitative analysis of survey data [36]. However, further investigation of historical population trends for all three of these possibly extinct species was conducted through an extensive interview survey conducted in fishing communities across the middle-lower Yangtze drainage [36]–[37], an area covering the entire late twentieth century range of the baiji and most of the recent historical ranges of the paddlefish and shad in this river system [24], [38]–[39]. Our standardized survey data on sighting records for baiji, paddlefish, shad and other species permit comparison of relative patterns of population decline shown by river dolphins and economically important fishes during the late twentieth and early twenty-first centuries, providing one of the first analyses of species abundance data derived from local ecological knowledge [40]. These data demonstrate that the status of apex predators can provide a meaningful surrogate for the status of other species with differing life histories and ecological roles in heavily degraded ecosystems.

## Results

**Figure 1:**
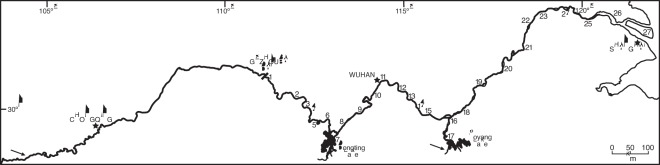
Middle-lower Yangtze drainage, showing distribution of interview localities (1–27), and key spawning grounds of Yangtze paddlefish (above Chongqing) and Reeves’ shad (Ganjiang River draining into Poyang Lake).

Interviews were conducted in 2008 in 27 fishing communities along the middle-lower Yangtze channel (1767 km stretch from Yichang downstream to the Yangtze estuary) and around two major lake systems (Dongting and Poyang Lakes) connected to the Yangtze mainstem (Figure 1; Supporting Information Table S1). Baiji, paddlefish and shad were all historically present over the entire survey area. A total of 599 informants were interviewed during the survey.

**Figure 2:**
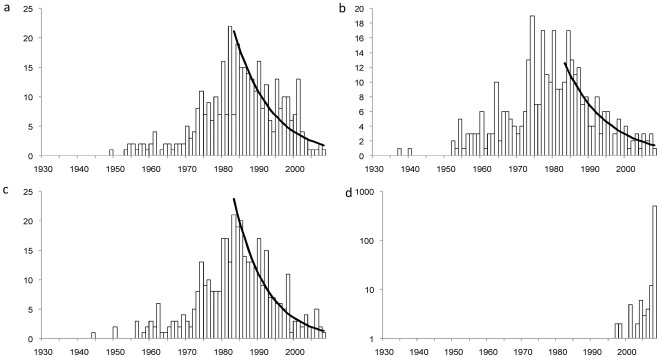
Frequency distribution (black bars) of last-sighting dates over time reported for (a) baiji, (b) Yangtze paddlefish, (c) Reeves’ shad, and (d) Yangtze finless porpoise, overlaid with back-transformation of linear regression (black line) to demonstrate the fit of our regression model to the original data. Porpoise last-sighting dates are displayed on a logarithmic scale to better illustrate the data.

**Figure 3:**
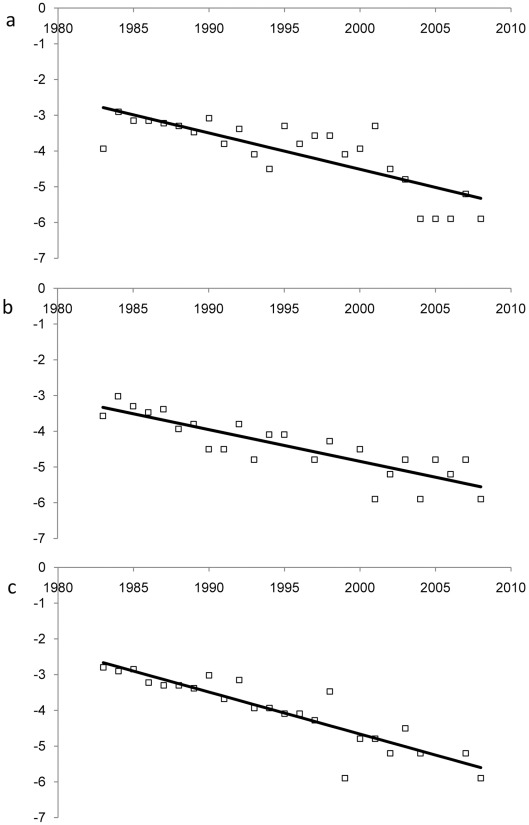
Transformed data showing results of comparative linear regression analysis for (a) baiji, (b) Yangtze paddlefish, and (c) Reeves’ shad.

**Figure 4:**
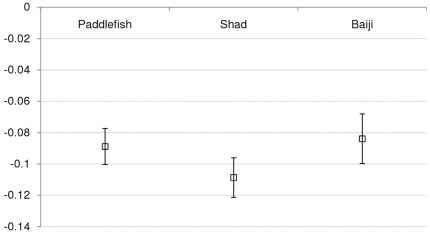
Comparison of gradients of transformed declines of baiji, Yangtze paddlefish and Reeves’ shad, showing close similarity between all three decline gradients.

In total, 365 baiji records, 338 paddlefish records and 375 shad records (single sightings reported by separate informants when asked to state the most recent year that they had seen each species, and identified as representing separate events due to differences in date, location, and/or other details) were collected for comparison of last-sighting dates. Untransformed last-sighting date frequency distributions for all three species show similar decline curves over time (Figure 2a-c). The mode for last sighting date was 1982 for baiji, 1983 for shad, and 1974 for paddlefish, although there were also three other, only slightly lower, peaks in paddlefish last-sighting date frequency between 1977 and 1984 (Figure 2b; the distribution of last-sighting dates for this species appears stable across this time period). The declines of all three species were therefore closely correlated in time, although the decline of the paddlefish may have started slightly earlier. The logistic decline shown by shad last-sighting dates is slightly steeper than shown by baiji or paddlefish (Figures 3–4), but the linear gradients of transformed last-sighting date series for all three species are not significantly different from each other (GLM species*year interaction: *F* = 0.94, *p* = 0.395), demonstrating that the three target species experienced statistically similar rates of population decline over time. The intercepts of the linear gradients of transformed last-sighting date series for all three species are also not significantly different (*F* = 0.95, *p* = 0.391). The non-significant result was also borne out by non-parametric Monte Carlo tests: variance test *p* = 0.643; minimum difference *p* = 0.144; middle difference *p* = 0.669; maximum difference *p* = 0.346.

Only 7.5% of informants who provided information on finless porpoise sightings (n = 563) stated that they had not seen the species in the year that interviews were conducted and provided pre-2008 last-sighting dates, and only a further 2.5% suggested they had not seen any porpoises in that year but were uncertain about when they had last seen the species (Figure 2d). It is not possible to formally analyze the porpoise data in a similar manner to those of baiji, paddlefish or shad, because only the later decline part of a last-sighting date frequency distribution is informative for understanding the dynamics of species decline and extinction (see Materials and Methods). As porpoises are still encountered on a fairly regular (at least annual) basis by most fishers, the decline part of the curve is not yet apparent, and the frequency distribution for porpoises contains few dates earlier than 2008 (the year the interview survey was conducted). However, the marked difference in last-sighting date frequency distribution for porpoise in comparison to the other study species (Figure 2) demonstrates that different sympatric species in the Yangtze system have experienced differential patterns of population decline.

## Discussion

Population trends are typically analysed using longitudinal studies that collect progressive data on the status of target taxa over time, a technique that can permit reconstruction of relative or absolute population status depending on data quality [41]. Our population trend data, in contrast, are compiled retrospectively using informant recollections of last-sighting dates obtained from interviews. This technique does not provide data on absolute population abundances over time, but permits comparison of relative population dynamics between different species. Although temporal distributions of last-sighting dates have previously been analysed within probabilistic frameworks to investigate other aspects of the extinction process (e.g. using optimal linear estimation to provide statistical constraints on extinction dates [36], [42]), few studies have so far explored the usefulness of interviews to monitor trends in threatened or exploited species [43], and to our knowledge interview-based informant data have not previously been used to construct single-species or multi-taxon decline curves. Interview surveys can represent a relatively inexpensive approach for collecting data across wide geographical areas. Although the lack of useful information on porpoise decline provided by last-sighting dates shows that this type of data is not necessarily appropriate for studying declining species that are still encountered relatively regularly, we suggest that our novel interview-based approach has much wider applicability for research into the population dynamics of those species that are both highly threatened, cryptic, or recently extinct, and also easily identifiable by untrained observers.

In addition to providing extensive new information on the extinction chronologies of baiji, paddlefish and shad, our last-sighting data demonstrate that these three species experienced correlated declines in terms of both the rate and timing of decline. This provides the first quantitative evidence that the status of dolphin populations can act as a population trend indicator of some other sympatric species, and perhaps of wider aquatic biodiversity. In order to assess the extent to which apex predators such as river dolphins can represent meaningful population trend indicators across other ecological systems, it is necessary to consider the relative significance of biological, ecological, and anthropogenic factors in determining patterns of extinction risk in freshwater taxa.

Historical data on trophic ecology and landscape-level habitat use of baiji, paddlefish and shad are limited [35], making it difficult to characterize their former ecological interrelationships. However, these species display markedly different life-history strategies, body masses and other key intrinsic parameters, suggesting that they are unlikely to have shared close ecosystem linkages. Later maturation and larger body size are both key intrinsic biological characteristics associated with higher extinction risk in marine fishes [44] and mammals [2]. These species might therefore be expected to show differing population responses to anthropogenic threats, so that it may seem surprising that a river dolphin can act as a meaningful population trend indicator for either fish species. However, the relationship between life history, body size and extinction risk in freshwater fishes is not straightforward, with smaller-bodied species also often highly threatened [45]–[46]. It has been suggested that the relative lack of unifying extinction-promoting biological/ecological traits in freshwater fishes indicates that human alterations to global freshwater habitats are already so severe that intrinsic factors have become less significant, with freshwater fish extinctions now primarily driven by extrinsic factors [47]. Indeed, the combined effects of pollution, overfishing, flow regulation, dense boat traffic and sedimentation, associated with escalating overpopulation and industrialization, have made the Yangtze drainage one of the world’s most degraded ecosystems [48]. It is therefore possible that it now represents a system in which the “field of bullets” model of extinction selectivity, where extinction is effectively unpredictable in relation to life history traits, is likely to apply [47], [49].

If the entire Yangtze freshwater biota has experienced a similar pattern of decline due to this overwhelming suite of extrinsic extinction drivers, the correlated declines of river dolphins and various fishes would be unsurprising and of little interest. However, both our porpoise last-sighting data and available Yangtze fisheries data [23] indicate that although all megafaunal species and economically important fishes have experienced some degree of population decline, different species have declined at markedly different rates and many species continue to be encountered and caught on a regular basis. We would therefore not necessarily expect the status of an apex predator in this system to bear any close relationship to that of any particular fish species. In order to better understand why baiji can act as a population trend indicator for paddlefish and shad, and to assess the wider potential for using river dolphins as surrogates of sympatric biodiversity, it is therefore necessary to identify specific factors that may determine population resilience in the Yangtze freshwater biota.

One key factor likely to explain at least some of the increased extinction vulnerability shown by baiji, paddlefish and shad in contrast to finless porpoise and other species is migratory status. Migratory reproductive behaviour is associated with higher extinction risk in fishes [50], because even if migratory species occur seasonally over large areas, they may have geographically restricted spawning grounds that are easily impacted by anthropogenic processes such as damming. Large-bodied non-migratory species such as common carp (*Cyprinus carpio*) and southern catfish (*Silurus meridionalis*) have remained relatively stable and widely distributed over much of the Yangtze drainage, and have become increasingly important target species for regional fisheries as other species have declined [23], [51]. Conversely, migratory and semi-migratory fishes (both potadromous and diadromous species) have experienced far greater declines across the Yangtze drainage, often resulting in regional extirpation [22]–[23], [51]. Both paddlefish and shad were diadromous species that underwent major upstream migrations to reach specific spawning grounds, including the Ganjiang River draining into Poyang Lake for shad [28], and the river section above Chongqing for paddlefish [24] (Figure 1). The Ganjiang River was cut off from Poyang Lake and the Yangtze mainstem by the Wanan Dam in 1984, and Chongqing was cut off by the Gezhouba Dam in 1981 [52]–[53]. Similarly, although the differences in extinction vulnerability between baiji and finless porpoise may also be associated with body size, life history, habitat requirements and/or feeding ecology (possibly influencing vulnerability to fishing gear entanglement [54]), baiji are known to have made large-scale seasonal upstream and downstream movements [36], whereas genetic data suggest that porpoises instead show strong site-fidelity [55]. Furthermore, in addition to their increased vulnerability due to migratory status, paddlefish and shad also experienced additional pressures from overexploitation; shad catches from the 1960s to the 1980s shifted progressively towards younger age classes, indicating severe recruitment overfishing [28], and overfishing also drove substantial stock depletion and body size decrease in paddlefish since at least the 1970s [56]. Both of these fish species may therefore have been particularly at risk from regional anthropogenic activities, due to their high vulnerability to two separate threat processes.

Even in an ecosystem such as the Yangtze that is exposed to a potentially overwhelming suite of extrinsic threats, different species can therefore be seen to respond differently to anthropogenic processes. However, our data suggest that the most vulnerable set of species (e.g. migratory species, and/or those species severely threatened by more than one different extinction driver) can experience correlated responses to extrinsic threats and display spatially and temporally similar patterns of decline, even if they represent otherwise very different taxonomic, biological and ecological groupings. The ability of temporal baiji population trend data to provide a meaningful indication of simultaneous trends in paddlefish and shad populations therefore does not represent a causal relationship associated with the baiji’s status as apex predator or putative keystone species, but instead the shared status of these species as being similarly highly vulnerable to regional threat processes due to specific aspects of their ecology (e.g. undertaking seasonal migrations). Conversely, finless porpoise population trend data do not represent a good surrogate for either paddlefish or shad population trends, because this species was less vulnerable to anthropogenic disturbance as a result of these specific ecological characteristics.

Monitoring the status of river dolphins or other charismatic and vulnerable freshwater megafauna has the potential to provide wider information on the status of other highly threatened components of sympatric freshwater biotas. However, whereas river dolphins may behave as indicators of the status of wider trends in biodiversity under such conditions, other species with a variety of different biological and ecological traits may thus also be expected to perform well, and may even be easier to monitor in practice [12]. Because population trends shown by baiji and finless porpoises have different relationships to population trends shown by highly vulnerable Yangtze fish species, freshwater cetaceans in other systems may also potentially display differing ecological relationships with the status of other sympatric taxa. Further research into the relationship between the status and trends shown by river dolphins and key fishes in freshwater environments exposed to differing intensities of human impact (e.g. Ganges River dolphins and migratory hilsa shad *Tenualosa ilisha* in the Ganges-Brahmaputra-Meghna drainage) is necessary to test the generality of these findings, to define patterns of ecological causality and correlation between these taxa, and to assess the wider usefulness of dolphins as indicators of the status of regional biodiversity and the ‘health’ of aquatic ecosystems.

## Materials and Methods

### Interview Survey

Informants were located through targeted sampling [57] by the authors (or with assistance from regional fisheries officials) in known fishing settlements, typically around fishing harbours or on moored boats, and sometimes when they were returning from fishing activities or when still on the river (latter interviews conducted by boat). It is possible that this sampling approach may have introduced some sampling bias into our survey; however, we consider that the subset of fishers we interviewed are likely to be broadly representative of the wider Yangtze fisher community in socio-economic background and ecological experience, as this interviewed subset includes informants with a wide range of variation in age and fishing practices [36]–[37]. Families often live and work together in fishing boats on the Yangtze, and so may share similar histories of experiencing different target species; only one family member was interviewed from each fishing family to remove this source of non-independence. All informants were interviewed on a one-to-one basis in relaxed informal settings by a native Chinese speaker, who followed a standard anonymous questionnaire containing descriptive, structured and contrast questions that took c. 30 minutes to complete (Text S1). Project staff remained neutral during interviews and avoided using leading questions to minimize the chance of influencing informant responses. The questionnaire and interview protocols were field-tested during a three-day pilot survey at Hukou (Jiangxi Province) to identify any problems arising during interviews, to improve clarity of questions, and to provide a training period for all interviewers; a second sample of fishers were later interviewed at Hukou during the main survey to ensure comparable data collection from this locality. Almost all informants completed the entire questionnaire.

As part of a wider series of interview questions (Text S1), informants were asked whether they had seen/caught baiji, paddlefish and shad; the date of their most recent sightings of each species; whether they knew of other recent sightings of these species by other people; and also about the frequency of their Yangtze finless porpoise sightings. Photographic cue cards of live wild/captive baiji, dead baiji, and captured/stranded paddlefish ([35], [58]–[60], or provided by the Institute of Hydrobiology and the Yangtze River Fisheries Research Institute) were shown to all informants to test their accurate identification of these species and the validity of their responses. Special care was taken to verify sighting records from the mid-1990s onwards, with informants required to provide a detailed description of their sighting that was not accepted unless it contained key diagnostic characteristics (e.g. white colour and/or long beak for baiji). Particular care was also taken to ensure that paddlefish was not confused with either of the other two native acipenseriforms (Chinese sturgeon *Acipenser sinensis*, Yangtze sturgeon *A*. *dabryanus*) also found in the middle-lower Yangtze. Any appropriate regional names for paddlefish (e.g. *baiqing*, *huangyu*, *qingqiangyu*, *qingyupaozi*, *qinyu*) recognized by at least one member of each fishing community were used in conjunction with the standard Chinese name for the species (*baixun*), and informants were required to demonstrate their ability to differentiate between these sympatric species (e.g. by describing key diagnostic characteristics of the paddlefish, such as its elongated rostrum) in order for their accounts to be recorded. Other data collected during the wider interview survey have been published elsewhere [36]–[37].

### Ethics Statement

Interview survey methods followed the Zoological Society of London (ZSL)’s guidelines for ensuring appropriate ethical standards in projects involving data collection from humans for research purposes, and standard fieldwork protocols for our longer-term research programme into local ecological knowledge in Yangtze communities have been approved by ZSL’s Ethics Committee (although these were not separately re-approved for this specific project). All informants were told about the aims of the survey and ensured that data would be analyzed anonymously; interviews were only conducted following verbal consent and approval of participants, such that their co-operation in completing an interview questionnaire represents our record of their verbal consent.

### Analysis

Although about half of all last-sighting dates (51.7% of baiji records, 45.6% of paddlefish records, 46.1% of shad records) were reported as direct calendar years, many informants also reported last-sighting dates in alternative formats: paired consecutive calendar years (e.g. ‘1986/1987’), decadal or other ranges (e.g. ‘1980s’, ‘late 1980s’), or decades/half-decades before 2008 (e.g. ‘20 years ago’, ‘25 years ago’). More recent sighting dates were more likely to be reported as direct calendar years because informants had more accurate recall over shorter time intervals, but to avoid excluding large amounts of additional sighting data from further analysis, dates reported in alternative formats were randomized to direct calendar years as follows: paired calendar years were given an equal probability of being assigned to either consecutive year; decadal or other specific ranges were given an equal probability of being assigned to any calendar year within this range; and dates rounded to a given number of decades or half-decades before 2008 were given an equal probability of being assigned to any calendar year ±5 the given value (e.g. ‘20 years ago’ represents a potential date range from 1983–1993).

The number of reported last-sighting dates for a recently extinct species is expected to decrease over recent time as a result of the species’ population decline, but will also drop off further back in time into the period before informants experienced their final sighting of the species. A frequency distribution of last-sighting dates will therefore be approximately bell-shaped (Figure 2a-c). Only the later decline part of this frequency distribution is informative for understanding the dynamics of species decline and extinction. In order to compare relative patterns of decline in baiji, paddlefish and shad, we therefore examined time series that were post-peak for all three species (from 1983 onwards; see Results). The frequency of last-sighting dates for each species for each year from 1983–2008 was expressed as a proportion of the total number of observations, and then subjected to a logistic transformation. The transformed data (response) were then linearly regressed on year (predictor) using a GLM command in Minitab version 16.1.0, and the regression slopes between species were compared using an interaction term in the model. The model’s word-equation can be written as: GLM [transformed last-sighting frequency]  =  year|species, where year was specified as a continuous variable.

As the residuals from the above model were slightly non-normal, and subsequent transformations did not correct this, they were also analyzed using a non-parametric Monte Carlo test of the differences between gradients. Several metrics of the real differences between gradients were compared with a set of values for each metric expected under the null hypothesis. These metrics (test statistics) comprised: variance among gradients; maximum value of the two-way difference between gradients in the three gradient pairs; and middle and minimum values of the difference between gradients in the three gradient pairs. The null values were generated as follows: for each year from 1983–2008, the values of the response for each species were randomly reassigned without replacement to one of the three species. Decline gradients were calculated in the same way as for the real data, for 99,999 randomizations. Test statistics for real species data were ranked within those for 99,999 randomly allocated data points to give *p* values.

Spatial variation in last-sighting dates across the survey area was not further investigated, because previous analysis of interview data has shown that baiji population decline was not associated with any major contraction in geographical range across the middle-lower Yangtze drainage [36].

## Supporting Information

Text S1 Yangtze fishermen interview questionnaire (English-language version).
(DOCX)

Table S1 Summary of interview data collected from the middle-lower Yangtze region.
(DOCX)
