# Supplementary material for: River Dolphins Can Act as Population Trend Indicators in Degraded Freshwater Systems
Source: PLoS One. 2012 May 29;7(5):e37902. doi: 10.1371/journal.pone.0037902 (PMC3362568; doi:10.1371/journal.pone.0037902)
Supplement: Text S1 — Yangtze fishermen interview questionnaire (English-language version). (DOCX) [file pone.0037902.s001.docx]

**Text S1: Yangtze fishermen survey questionnaire**

**(English-language version)**

DATE: LOCATION: INTERVIEWER:

**A: FISHERIES QUESTIONS**

1) Are you a professional fisherman?

a) Are you retired?

2) How old are you?

3) How many years have you been fishing?

4) What kind of fishing gear do you use today? (list all if more than one type)

Free-floating gill nets Hao wang

Drag nets (feng wang) Si wang

Drag nets (wei wang) Shrimp traps

Gill net / drag net (tuo wang) Crab net (xie wang)

Gill net (san ceng wang) Rolling hooks

Other type of fishing gear (describe)

5) Size dimensions of gill / drag net:

a) Mesh size

b) Net length

c) Net width

6) Have you always used this kind of fishing gear? (Y/N)

a) When and why did you change your fishing gear?

7) What is the commonest type of fishing gear used in your village?

8) Have you ever used rolling hooks in the past? (Y/N)

a) When did you stop using them?

9) Do you know how many people still use rolling hooks in this section of the

river? (Y/N)

a) How many?

10) Do you ever lose/have to replace your fishing gear? (Y/N)

a) How often?

11) Do you ever see ‘ghost’ fishing gear in the river? (Y/N)

a) What kind?

b) How many times in the past year?

12) Is there a problem with electro-fishing around here? (Y/N)

a) Do you know how many electro-fishermen there are in this river

section?

b) How many years ago did electro-fishing start in this river section?

13) What species of fish do you catch?

1. Japanese eel *Anguilla japonica* (manli)
2. Crucian carp *Carassius carassius* (jiyu)
3. Predatory carp *Chanodichthys erythropterus* (boyu)
4. Grenadier anchovy *Coilia nasus* (fengweiyu)
5. Grass carp *Ctenopharyngodon idella* (caoyu)
6. Common carp *Cyprinus carpio* (liyu)
7. Sharpbelly *Hemiculter leucisculus* (cantiaoyu)
8. Silver carp *Hypophthalmichthys molitrix* (lianyu)
9. Bighead carp *Hypophthalmichthys nobilis* (yongyu)
10. Chinese longsnout catfish *Leiocassis longirostris* (huiyu)
11. Wuchang bream *Megalobrama amblycephala* (fangyu)
12. Black carp *Mylopharyngodon piceus* (qingyu)
13. Yellowhead catfish *Pelteobagrus fulvidraco* (huangsangyu)
14. Southern catfish *Silurus meridionalis* (nianyu)
15. Chinese perch *Siniperca chuatsi* (guiyu)
16. Other species (list/describe)

14) Have you ever seen or caught Reeves’ shad (shiyu)? (Y/N)

a) When did you last see this fish?

b) When is the last time that anybody saw this fish?

15) How many hours do you spend on the river each day?

16) How many days a week do you go fishing?

17) What time of day or night do you go fishing?

18) How much time is your fishing gear in the water for each week?

19) What job do you do during the fishing ban?

20) Apart from the fishing ban, do you do different amounts of fishing at

different times of year? (Y/N)

a) Which months do you do the most fishing?

b) Which months do you do the least fishing?

21) Where do you fish?

Main channel (mid) **OFTEN SOMETIMES RARELY**

Main channel (near bank) **OFTEN SOMETIMES RARELY**

Behind sandbars/islands **OFTEN SOMETIMES RARELY**

In tributaries **OFTEN SOMETIMES RARELY**

22) What are the upstream and downstream boundaries of where you go

fishing?

23) Have you always fished in this region? (Y/N)

a) Where did you used to fish?

b) When did you change your fishing range?

24) What kinds of changes have you noticed over time?

a) Amount of fish caught:

**BETTER / SAME / WORSE THAN BEFORE**

b) Declines of particular species (name any species that have declined)

c) Number of fishing boats on the river:

**MORE / SAME / FEWER THAN BEFORE**

25) Do you want your children to be fishermen? Is it a good job for the next

generation? (Y/N)

*SPACE FOR ADDITIONAL COMMENTS:*

**B: FINLESS PORPOISE QUESTIONS**

[Informant is asked to identify a finless porpoise from a series of photographs without being prompted. If he/she is able to do this, the following questions are asked.]

26) How often do you see porpoises when you are on the river?

27) Do you see more porpoises at a particular time of year? (Y/N)

a) When?

28) How many porpoises do you normally see at a time?

29) Do you think that there are as many porpoises today as there

used to be in the past? (Y/N)

30) Do porpoises ever take fish out of your nets? (Y/N)

31) Have you ever seen dead porpoises, or heard about

porpoises getting killed? (Y/N)

a) When did you last see a dead porpoise? (month, year)

b) Where was this animal seen?

c) Do you know what killed the porpoise?

d) Do you have any information on any other dead porpoises?

32) Do you know if there are any dead porpoises around here anywhere? (Y/N)

33) Do porpoises ever get killed by ships?

1. When was the last time this happened?
2. How often does this happen?
3. How many times has this happened in the last five years?
4. Where in the river do ships kill porpoises?

i. Distance from bank:

ii. Geographical location:

1. How do you know it that the porpoise was killed by a ship?

34) Do porpoises ever get killed by electro-fishing? (Y/N)

1. When was the last time this happened?
2. How often does this happen?
3. How many times has this happened in the last five years?

35) Do porpoises ever get killed in fishing gear? (Y/N)

a) What kind of fishing gear? (describe mesh size)

1. When was the last time this happened?
2. How often does this happen?
3. How many times has this happened in the last five years?
4. Where in the river does fishing gear kill porpoises?

i. Main channel, side-channel or tributaries:

ii. Distance from bank:

iii. Geographical location:

36) If porpoises get caught in fishing gear, what do people do with them?

37) In what months/seasons do porpoises most commonly get caught/killed?

*SPACE FOR ADDITIONAL COMMENTS:*

**C: BAIJI QUESTIONS**

[Informant is asked to identify a baiji from a series of photographs without being prompted. If he/she is able to do this, the following questions are asked.]

38) Do you know what a baiji is? (Y/N)

39) Have you ever seen a baiji in your lifetime? (Y/N)

a) How many times have you ever seen baiji?

1. When was the last time you saw a baiji?
2. Where was the last place you saw a baiji?
3. Do you remember what you were fishing for when you saw baiji?

i. What species were you fishing for?

40) Have you ever seen more than one baiji at a time?

a) What is the largest group of baiji you have seen?

1. Where did you see this group?
2. When did you see this group?
3. Do you remember what you were fishing for when you saw baiji?

i. What species were you fishing for?

41) Describe your baiji sightings (e.g. duration, habitat, behaviour, time of

year, distance from boat).

42) If you have never seen a baiji, how have you heard about the species?

43) Do you know of anyone else that has ever seen a baiji? (Y/N)

a) Describe their baiji sightings (e.g. location, date, duration, habitat,

behaviour, time of year, distance from boat).

44) Do you know of anyone specific who we might want to talk to, who might

know more about baiji? (Y/N)

45) Have you ever seen a dead baiji? (Y/N)

a) How many dead baiji have you seen?

b) Describe any dead baiji that were seen (location, date, other

details).

46) Have you ever heard of anyone catching a baiji? (Y/N)

a) If so, what kind of fishing gear it was caught in?

47) Have you ever heard of baiji being killed in any other way? (Y/N)

(e.g. strandings, boat collisions)

a) Describe:

48) If baiji ever got caught in fishing gear, what happened to them?

49) Did you notice any particular period in time when baiji became much

rarer? (Y/N)

a) When?

50) When do you think baiji disappeared?

51) What do you think caused the baiji to disappear?

52) Why do you think that the porpoise has survived but the baiji has

disappeared?

53) Do you know about any stories, myths or legends about the baiji?

*SPACE FOR ADDITIONAL COMMENTS:*

**D: PADDLEFISH QUESTIONS**

[Informant is asked to identify a paddlefish from a series of photographs without being prompted. If he/she is able to do this, the following questions are asked.]

54) Do you know what a paddlefish is? (Y/N)

55) Have you ever caught a paddlefish in your lifetime? (Y/N)

a) How many paddlefish have you ever caught?

1. When was the last time you caught a paddlefish?
2. When is the last time that anybody you know caught a paddlefish?
3. Where was the last place you caught a paddlefish?
4. What kind of fishing gear were you using, and where in the river?

56) If you have never caught a paddlefish, how do you know about the species?

57) Do you know anyone else that has ever seen or caught a paddlefish? (Y/N)

a) Describe the sighting (date, location, other details):

58) Do you know of anyone specific who we might want to talk to, who might

know more about paddlefish? (Y/N)

59) Have you ever heard of paddlefish being killed in any other way? (Y/N)

(e.g. strandings, boat collisions)

1. Describe:

*SPACE FOR ADDITIONAL COMMENTS:*
