# Supplementary material for: River Dolphins Can Act as Population Trend Indicators in Degraded Freshwater Systems
Source: PLoS One. 2012 May 29;7(5):e37902. doi: 10.1371/journal.pone.0037902 (PMC3362568; doi:10.1371/journal.pone.0037902)
Supplement: Table S1 — Summary of interview data collected from the middle-lower Yangtze region. (DOCX) [file pone.0037902.s002.docx]

**Table S1**

Summary of interview data collected from the middle-lower Yangtze region. Interview data were collected approximately evenly across the middle-lower Yangtze channel transect and its major appended lakes.

| **Interview locality** | **Downstream distance from Yichang** | **Number of interviews** |
| --- | --- | --- |
| Yichang | 0 | 31 |
| Longzhou | 130 | 20 |
| Jianglin | 190 | 12 |
| Xinchang | 213 | 4 |
| Shishou | 238 | 27 |
| Jianli | 313 | 18 |
| Yueyang | In Dongting Lake | 30 |
| Honghu | 445 | 5 |
| Jiayu | 495 | 42 |
| Jinkou | 595 | 9 |
| Wuhan | 626 | 30 |
| Ezhou | 722 | 6 |
| Huangshi | 760 | 6 |
| Qizhou | 793 | 19 |
| Wuxue | 830 | 31 |
| Hukou | 901 | 41 |
| Douchang | In Poyang Lake | 30 |
| Pengze | 936 | 15 |
| Anqing | 1030 | 35 |
| Tongling | 1123 | 35 |
| Wuhu | 1226 | 21 |
| Wujiang | 1290 | 17 |
| Nanjing | 1321 | 37 |
| Zhenjiang | 1404 | 28 |
| Jiangyin | 1512 | 15 |
| Nantong | 1570 | 15 |
| Chongming | 1650 | 20 |
